# Longitudinal Analysis of Self-Reported Symptoms, Behavioral Measures, and Event-Related Potential Components of a Cued Go/NoGo Task in Adults With Attention-Deficit/Hyperactivity Disorder and Controls

**DOI:** 10.3389/fnhum.2022.767789

**Published:** 2022-02-18

**Authors:** Marionna Münger, Silvano Sele, Gian Candrian, Johannes Kasper, Hossam Abdel-Rehim, Dominique Eich-Höchli, Andreas Müller, Lutz Jäncke

**Affiliations:** ^1^Division of Neuropsychology, Institute of Psychology, University of Zurich, Zurich, Switzerland; ^2^University Research Priority Program (URPP) “Dynamics of Healthy Aging”, Zurich, Switzerland; ^3^Brain and Trauma Foundation Grisons, Chur, Switzerland; ^4^Praxisgemeinschaft Psychiatrie und Psychotherapie, Lucerne, Switzerland; ^5^Psychiatrie und Psychotherapie Rapperswil, Rapperswil, Switzerland

**Keywords:** attention-deficit/hyperactivity disorder (ADHD), event-related potentials (ERPs), adults, reliability, longitudinal study, Go/NoGo task, continuous performance test (CPT), electroencephalography (EEG)

## Abstract

This study characterizes a large sample of adults with attention-deficit/hyperactivity disorder (ADHD) and healthy controls regarding their task performance and neurophysiology; cross-sectionally and longitudinally. Self-reported symptoms, behavioral measures, and event-related potentials from a classical cued Go/NoGo task were used to outline the symptom burden, executive function deficits and neurophysiological features, and the associations between these domains. The study participants (*N* = 210 ADHD, *N* = 158 controls, age: 18–62 years) were assessed five (ADHD) or three (controls) times over two years. We describe cross-sectional and longitudinal group differences, and associations between symptom burden, and behavioral and event-related potential (ERP) components variables by latent growth curve models, including random slopes and intercepts. The ADHD group showed increased reaction time variability, increased commission and omission errors, and attenuated cueP3, CNV, N2d, and P3d amplitudes. We observed a decrease in self-reported symptoms in the ADHD group over the two years. The behavioral measures (reaction time variability, number of omission, and commission errors) did not change over time, whereas the cueP3, P3d, and N2d amplitude attenuated in both groups. There was no evidence for a robust association between symptom burden and behavioral or ERP measures. The changes in the ERP components with stable task performance, potentially indicate more efficient neuronal processing over the two years. Whether the lack of association between symptom burden and behavioral or ERP measures might be due to the low reliability of the ADHD assessment criteria, or the inappropriateness of the objective measures cannot be inferred.

## Introduction

Attention-deficit/hyperactivity disorder (ADHD) is a neurodevelopmental disorder diagnosed based on the clinical examination. The symptoms are currently assessed and quantified based on the fifth edition of the Diagnostic and Statistical Manual of Mental Disorders (DSM-5) (American Psychiatric Association [APA], 2013). According to the DSM-5, individuals with ADHD “demonstrate a persistent pattern of inattention and/or hyperactivity-impulsivity interfering with functioning or development.” The manifestation of the disorder can be assigned to one of three categories: predominantly inattentive presentation, predominantly hyperactive/impulsive presentation or as combined presentation (American Psychiatric Association [APA], 2013). For adults, the diagnostic criteria are met, if five out of the nine symptoms are present. Symptoms for *inattention* and *hyperactivity and impulsivity* are listed and evaluated separately. Symptoms listed in the inattention domain are for example: “…is often easily distracted by extraneous stimuli or unrelated thoughts”, “…often does not seem to listen when spoken to directly or fails to follow instructions”. Symptoms listed in the hyperactivity and impulsivity domain are for example: “…often leaves seat in situations when remaining seated is expected”, “…often has difficulty waiting for his or her turn or interrupts on others.” The diagnosis by DSM-5 is based on the number of observable symptoms. The overall symptom burden is mostly quantified by the number of symptoms and their frequency of occurrence in daily life (van Lieshout et al., 2019; Nylander et al., 2021).

In the context of empirical research on ADHD, deficiencies in executive functions have been identified and proposed as potential phenotype of the disorder (e.g., Sergeant, 2000; Sonuga-Barke et al., 2008; Kofler et al., 2019). These deficiencies are often linked to specific (mostly deficient) neurophysiological processes, supporting the idea of ADHD as a neurocognitive dysfunction. Inhibitory control, attention and working memory are those segments of executive functions, which are most prominently affected in ADHD.

Currently, two models are discussed to describe the underlying neurocognitive functions in ADHD subjects: the maturational lag hypothesis and the trait liability hypothesis. The maturational lag hypothesis states that the observed neurocognitive deficits in children with ADHD derive mainly from neurophysiological immaturity. Accordingly, affected children will partially or fully remit from the impairments and catch up with their peers by adulthood (Halperin and Schulz, 2006; Doehnert et al., 2010, 2013). This hypothesis contrasts deficient neurocognitive performance in adults with ADHD compared to healthy controls (Mowinckel et al., 2015; Nikolas et al., 2019). In opposition to the maturational lag hypothesis, the trait liability hypothesis assumes that the neurocognitive deficits are the core reason of the disorder (Barkley, 1997). It states that these impairments persist, independent of age and alteration in symptoms. Evidence for the liability hypothesis derives from longitudinal studies with children and adolescents which show that individuals with ADHD have persistent neurocognitive deficits (Albrecht et al., 2008; Gau and Shang, 2010; Leopold et al., 2019).

Longitudinal studies describing symptom burden, cognition (Alperin et al., 2017; van Lieshout et al., 2019; Tallberg et al., 2021), neurophysiology (Doehnert et al., 2010, 2013; Du Rietz et al., 2016; Lau-Zhu et al., 2019), and their association focus exclusively on children. Only a single study predicts long-term outcomes in adult ADHD based on data obtained during childhood and adolescents (Nylander et al., 2021). However, this study included only symptom burden, global functioning, and demographic information (medication, comorbidity, intelligence quotient, age, and sex), without considering neurophysiological characteristics of the disorder. Knowledge on the stability of neurophysiological measures, neurocognitive functional deficits, and its association with the symptom burden in adult ADHD is lacking.

The continuous performance test (CPT) is one of the most frequently used tests examining executive functions in ADHD subjects. A standard variant of CPTs are visually cued Go/NoGo tasks, using a two-stimulus paradigm in which the first stimulus serves as the cue and the second as the target. Some tasks require an active response by the participant. Such tests challenge the core characteristics of ADHD, which are sustained attention, inhibitory control, and stability of cognitive processing. On a behavioral level, the CPTs provide quantitative measures of executive function performance. Difficulties in sustained attention are reflected by increased omission errors (Losier et al., 1996; Metin et al., 2012), lack of inhibitory control by an increased number of commission errors (Losier et al., 1996; Metin et al., 2012), and unstable cognitive processing by increased reaction time variability (Karalunas et al., 2014).

The neurophysiological underpinnings during a CPT have been studied extensively in healthy adults using event-related potential (ERPs) components (Randall and Smith, 2011; Albert et al., 2013; Downes et al., 2017; Kropotov et al., 2017; de Tommaso et al., 2020). In addition, several studies have examined these ERP components in individuals with ADHD. A recent meta-analysis revealed attenuated amplitudes and increased latencies in several components (Kaiser et al., 2020). Most of these studies focus on components that address inhibition of action, sustained attention and conflict detection, and show that ADHD subjects moderately differ from healthy controls (Münger et al., 2021).

Among the most studied ERP components in ADHD research are the positive peak around 300 ms after the cue stimulus (cueP3), the negativity approximately 100 ms before the second stimulus named contingent negative variation (CNV), the negative component around 200 ms (N2), and the positive component around 300 ms (P3) after the target stimulus.

The first two components, the cueP3 and CNV, are related to attentional orienting and preparatory processes. The amplitude of the cueP3 component represents attentional orienting (Polich, 2007; Doehnert et al., 2010). Robust evidence for attenuated amplitudes of the cueP3 was found in children and adolescents with ADHD (Kropotov et al., 2011; Albrecht et al., 2013; Tye et al., 2014; Du Rietz et al., 2016; Rommel et al., 2017), with mixed results in adults (McLoughlin et al., 2010; Grane et al., 2016). The above-mentioned meta-analysis estimated a negative mean effect size and 95% confidence interval for the ADHD group of *d* = −0.56 [−0.82, −0.30] (Kaiser et al., 2020). Interestingly, these studies identified larger mean effect sizes in adults compared to children and adolescents, making this component especially interesting for adult ADHD research. The moderator analysis in the meta-analysis indicated especially large effect sizes in tasks that require high inhibitory control. The independence of the cueP3 amplitude from the IQ score corroborates that this component represents rather attentional than cognitive functionality.

The amplitude of the CNV component represents resource allocation for an upcoming target, and represents preparatory processes, including anticipatory attention (Walter et al., 1964; Brunia and Van Boxtel, 2001). Its contribution to action preparation is supported by the source analysis in fMRI data, showing activation of supplementary motor area during the CNV (Nagai et al., 2004). A recent meta-analysis revealed that the CNV is moderately attenuated in ADHD subjects with an effect size of 0.32 [0.03, 0.61] (Kaiser et al., 2020).

The N2 and P3 components, identified after the target stimulus, are related to cognitive and inhibitory control (Enriquez-Geppert et al., 2010; Huster et al., 2010; Kropotov et al., 2011, 2017; Downes et al., 2017). Early experiments have shown larger N2 components in the NoGo compared to the Go condition (Falkenstein et al., 1999; Woltering et al., 2013). Henceforth the N2 component is interpreted as a neurophysiological marker of inhibitory control. However, subsequent studies have shown a sensitivity of the N2 component to the frequency of required responses and started the discussion about the involvement of the N2 in conflict detection (Nieuwenhuis et al., 2003). Nonetheless, the role in inhibitory control is of great interest for ADHD, more so for the predominantly hyperactive/impulsive presentation. While some studies with adolescents found attenuated N2 amplitudes in ADHD subjects compared to controls (Woltering et al., 2013), studies with adults did not find group differences (Grane et al., 2016; Kropotov et al., 2019). This may indicate that inhibition is of lower importance in adult ADHD. The P3 component has been originally associated to classification processes and action inhibition (Falkenstein et al., 1993, 1999). There is additional evidence, that the P3 component is also representing task evaluation (Huster et al., 2013). Generally, the NoGo condition was proven to be more powerful to distinguish ADHD subjects from controls than the Go condition (Johnstone et al., 2013; Kaiser et al., 2020). The meta-analysis of Kaiser et al. found a medium effect size of *d* = −0.57 [−0.90 – (−0.24)] for the P3 attenuation in ADHD subjects.

Many researchers have used the difference waves between the Go and NoGo tasks (ERP obtained during Go minus ERP obtained during NoGo trials). These difference waves indicate elegantly the involved neurophysiological and psychological processes. Two prominent components of these difference waves have been proposed: (a) the fronto-central N2d and (b) the fronto-central P3d (Kropotov et al., 2019). Recent studies clearly show an association of the N2d with conflict detection and P3d with action inhibition (Enriquez-Geppert et al., 2010; Randall and Smith, 2011; Albert et al., 2013; Kropotov et al., 2019). The differences between individuals with and without ADHD in these studies emphasize the importance of conflict detection and inhibitory control for the disorder.

ERP data give valuable insights into the deviant cognitive processing of participants diagnosed with ADHD. However, the linkage between the present diagnostic procedure, which relies on symptom report as the determining variable, and the objective measure from the neurophysiological assessment is challenging. A few cross-sectional studies report associations between symptom burden and ERP measures (Woltering et al., 2013; Tye et al., 2014; Grane et al., 2016). These studies report small to moderate correlation coefficients between symptom burden and ERP measures (*r* ∼ 0.2–0.45). The large intraindividual difference in perceived and reported symptom burden may explain the low correlation in cross-sectional studies. Association between the within-subject changes over several assessments can control for factors which are challenging to control for, such as perceived cognitive load or stress elicited by the task.

Short-term test-retest reliability (over hours, days, or weeks) of the ERP components shows moderate to high levels of reliability in terms of intraclass correlation coefficients (Kompatsiari et al., 2016; Taylor et al., 2016; Behforuzi et al., 2019; Devos et al., 2020). This indicates that ERP amplitudes and latencies can be assessed accurately within a short period. However, it is known that ERP components change throughout the lifetime. Cross-sectional analysis suggest that during childhood maturation, ERP amplitudes increase, whereas ERP latencies decrease with age (Münger et al., 2021). This can be interpreted as a gain in function, where cognitive operations become more pronounced and faster with increasing age. This interpretation is supported by the decreasing number of omission errors and faster reaction times with age (Münger et al., 2021). In contrast, during adulthood ERP amplitudes decrease and latencies increase with age (Kropotov et al., 2016; Münger et al., 2021). The later and attenuated peaks are interpreted to reflect a slowing and decline in cognitive processes (Kropotov et al., 2016), which have previously been summarized in the cognitive speed hypothesis of aging (Salthouse, 1996).

However, these effects of age are present in individuals with ADHD and healthy controls. Longitudinal studies are required to disentangle normal maturation from potential deviations in subjects with ADHD. Only a few studies investigated the developmental effects of ERPs in children and adolescents with and without ADHD using a longitudinal design (Doehnert et al., 2010, 2013; Petersen et al., 2018). Doehnert et al. (2010, 2013) followed a cohort through childhood and adolescence, including a follow-up in early adulthood. The studies do neither provide clear evidence for nor against the maturational lag hypothesis. Generally, the maturational trajectory is similar for individuals with and without ADHD and most group differences persisted or even increased throughout adolescence (Doehnert et al., 2010). However, some group differences become non-significant at the follow-up assessment in adulthood, which can be interpreted as a support for the maturational lag hypothesis (Doehnert et al., 2013).

Nonetheless, the existence of adult ADHD emphasizes that the maturational lag hypothesis misses important aspect of the course of ADHD. We therefore used a sample of adults to examine alterations in symptom burden and neurophysiology over time independent of developmental changes.

As discussed above, there is a lack of comprehensive investigations of the alteration in neurophysiological processing, task performance, and symptom burden in adults with ADHD over time. Longitudinal analyses adjust for the interindividual variance, which limits the meaningfulness of cross-sectional results. We aim to explore the ADHD symptom burden, executive function performance and neurophysiology among adults with ADHD and healthy controls over two years. This further allows us to investigate if potential changes in perceived symptom burden are associated with alterations in behavioral and/or ERP measures of executive functions.

First, we re-evaluate the average group differences between participants with ADHD and controls at baseline in self-reported symptoms, behavioral measures, and ERP components of a cued Go/NoGo task. Second, we describe the change over time in the control group and potential deviations in the ADHD group of those variables (univariate models). Third, within the ADHD group, we investigate the association between the self-reported symptoms and the behavioral and ERP measures, considering person-specific variation (bivariate models).

In this observational study with an adult sample, we do not anticipate alleviated symptoms or improved task performance over two years on a group level. Following the trait liability hypothesis, we hypothesize that potential changes in behavioral and neurophysiological measures on an individual level are not associated with the ADHD symptom burden.

## Materials and Methods

### Study Design and Participants

The data was taken from a large multicenter clinical study of the Brain and Trauma Foundation Grison (Switzerland). This large database contains information from more than 674 participants. In the current study, we analyzed the data of adults for whom complete baseline demographical information was available. This resulted in a total of *N* = 368 participants (controls *N* = 158, ADHD *N* = 210), with an age range of 18–62 years. All participants were examined within a period of two years. During this period, the participants with ADHD were assessed five times separated at by a 6-month interval (t_1_, t_2_, t_3_, t_4_, and t_5_). The control participants were assessed three times with a 12-month interval (t_1_, t_3_, and t_5_). At each assessment, all participants completed a questionnaire to assess their symptom burden. EEG data was recorded during resting state (not reported here) and during a cued Go/NoGo task performance. In addition, we administered the Wiener Matrizen-Test 2 (WMT-2) to assess fluid intelligence at baseline and the end of the study (Formann et al., 2011).

The demographic data of the sample at the first assessment, specified within the two groups, is shown in Table 1. The sex ratio in the ADHD group was balanced (f/m ADHD = 0.96), whereas more female than male participants were enrolled in the control group (f/m controls = 2.16). The two groups differed slightly in terms of mean age and IQ {age: *t*(366) = −2.22, *p* = 0.027, *d* = 0.23 [0.03, 0.44], IQ: *t*(352) = 3.78, *p* < 0.001, *d* = −0.41 [−0.62, −0.19]}. All participants were medication free on the day of the assessment, albeit *N* = 74 (35%) of the ADHD group indicated use of methylphenidate for their daily routine at the first assessment [no use: *N* = 85 (40%), missing information: *N* = 51 (25%)].

**TABLE 1:** Demographic data at baseline (t_1_).

	Control	ADHD
*N* total	158	210
*N* (%), male	50 (32%)	107 (51%)
*N* (%), female	108 (68%)	103 (49%)
Age (years)	32.5 ± 12.0	35.1 ± 10.1
IQ	105 ± 14	99 ± 16

*The table lists for both groups the number of male and female participants and the means and standard deviations for age and IQ (Missing data for IQ; control: N = 6, ADHD: N = 8).*

Certified psychiatrists or clinical psychologists confirmed the ADHD diagnosis according to the DSM-5 at the first assessment. The ADHD subtype is determined by the self-reported symptom burden.

We addressed the differences in the sex-ratio and age at baseline between groups by including these variables in the structural equation models (for details, see “Statistical Analysis”). The participants described here are part of the total cohort described in a previous publication (Münger et al., 2021).

In the control group, 25 participants (16%) were lost to follow-up (*N* = 15 at t_3_, *N* = 10 at t_5_). In the ADHD group 63 participants (30%) dropped out (*N* = 33 at t_2_, *N* = 11 at t_3_, *N* = 13 at t4, *N* = 6 at t_5_). In addition, EEG assessment was missing for some participants at individual time points (control group: *N* = 3 at t_3_, ADHD group: *N* = 3 at t_2_, *N* = 9 at t_3_, *N* = 6 at t_4_, *N* = 2 at t_5_).

To check for potential bias of losing participants to follow-up assessments, we compared the baseline characteristics between subjects who completed the trial and those who dropped out. The categorical variables sex, medication intake and therapy were tested by Fisher’s exact test and the continuous variables were tested by Welch’s *t*-tests. Within the control group, there is no evidence for any difference between subjects who completed the trial (*N* = 133) and those who were lost to follow-ups (*N* = 25). Within the ADHD group, none of the categorical variables, sex, medication intake, and therapy, showed a difference between subjects who completed the trial (*N* = 148) and those who were lost to follow-ups (*N* = 62). For the continuous variables, we found evidence for group differences for age and the P3d component. ADHD subjects who were lost to follow-ups, were on average younger *t*(128) = −3.17, *p* = 0.002, Cohen’s *d* = −0.45 [−0.76, −0.15], had larger P3d amplitudes *t*(115) = 2.34, *p* = 0.021, Cohen’s *d* = 0.35 [0.05, 0.65] and shorter P3d latencies *t*(115) = −2.65, *p* = 0.009, Cohen’s *d* = −0.40 [−0.70, −0.10] then ADHD subjects which completed the trials. There was no evidence for a difference in IQ, ADHD symptom burden, or any of the behavioral measures of the VCPT measures (reaction time, reaction time variability, and the number of errors) between the ADHD subjects who completed the trial and those who dropped out. Details of the analysis for both groups are reported in the Supplementary Tables 5a,b.

The data was collected between 2014 and 2018 at five private clinics in Switzerland. The study was approved by Zurich’s cantonal ethics committee (LeitEKZH_2013-0327/EKNZ_2014_160). All participants gave written informed consent and did not meet any of the exclusion criteria: another primary mental disorder, traumatic brain injury or loss of consciousness in the past, current or past drug abuse, pregnancy, epilepsy, and IQ < 80.

### Self-Reported Symptoms and Attention-Deficit/Hyperactivity Disorder Presentation

Participants in both groups completed a questionnaire based on the DSM-5 to assess the ADHD-related symptom burden at each assessment time point. Each item of the symptom’s list was rated on a five-point Likert scale (0: never, 1: rare, 2: sometimes, 3: often, and 4: very often). The scores were summed separately for the two domains in the DSM-5 (ADHD inattention and ADHD hyperactivity), resulting in an ADHD inattention score and an ADHD hyperactivity score.

We followed the DSM-5 to determine the ADHD subtype of the participants in the ADHD group. Items rated with often or very often were dummy coded with 1. If the sum was 5 or higher within one domain, the participant was categorized either as predominantly inattentive presentation (ADHD-inattention) or predominantly hyperactive presentation (ADHD-hyperactivity). A participant who exceeded the threshold of both domains (ADHD-inattention and ADHD-hyperactivity) as classified as a combined ADHD presentation (ADHD-combined).

### Medication Intake and Attended Therapies

Each month, participants were asked to complete an online questionnaire about their general well-being, medication intake and attended therapies. The reported data were obtained in the months directly after the first assessment (t_1_) and in the last month prior to the final (t_5_) assessment.

Participants could select the medications and therapeutic activities from a list of potential interventions. The report of *menthylphenidate intake* considered the following medicines: Ritalin, Medikinet, Concerta, and Focalin. The type of antidepressants was not surveyed. The therapeutic interventions *psychotherapy* and *neurofeedback* were assessed separately, whereas a set of additional interventions was summarized in the category *others* (coping, support in the work environment, support group, and memory training).

### Go/NoGo Task

We applied an established visual continuous performance test (VCPT) (Grane et al., 2016; Müller et al., 2019; Ponomarev et al., 2019), which is a classical cued Go/NoGo task. The test lasts 22 mins and comprises 400 trials, each consisting of a pair of sequentially presented visual stimuli. The cue stimulus (S1) is presented at 300 ms after trial onset and the target stimulus (S2) at 1,400 ms. Each is daf presented for 100 ms with an inter-trial interval of 1,000 ms. The stimuli are pictures of animals, plants, or humans. The task consists of four conditions: Go, animal-animal; NoGo, animal-plant; ignore, plant-plant; and ignore with auditory stimulus, plant-human. All conditions are presented equally often (25%, or 100 trials). The conditions are presented in a pseudo-randomized order. In the Go conditions, participants are asked to press a button, while in the NoGo condition participants must refrain from pressing the button. All trials beginning with the presentation of a plant stimulus could be ignored and did not require any action. The neurophysiological response to the two ignore conditions are not examined in this study. The task is described in detail in other publications; see for example Grane et al. (2016).

As behavioral measures, we assessed the mean reaction time, reaction time variability (coefficient of variance = mean reaction time/standard deviation of reaction time), the number of omission errors in Go trials (button was not pressed) and the number of commission errors in NoGo trials (button was pressed).

As neurophysiological measures, we looked at the amplitudes and latencies of four previously described and commonly used ERP components in ADHD research: cueP3, CNV, N2d, and P3d (Grane et al., 2016; Müller et al., 2019; Ponomarev et al., 2019; Kaiser et al., 2020). The presentation of an animal as the cue stimulus indicates a potentially upcoming Go stimulus, requiring an active button press and puts the participant in a state of increased alertness. In these trials (animal-animal or animal-plant), we looked at the positive component after the cue stimulus (cueP3-Pz), the negative component prior to the second stimulus (CNV-Cz, contingent negative variation), and the negative and positive component after the second stimulus for which we computed the difference curve between the NoGo and Go trials (N2d-Cz and P3d-Cz). All ERP components were measured at the leads where the waves are known to be most prominent (Grane et al., 2016; Cheung et al., 2017; Lau-Zhu et al., 2019).

### Electroencephalography Assessment and Processing

Electroencephalography (EEG) signals were acquired by a 19-channel tin electrode cap (standard 10–20 placement system) with two reference electrodes attached to the earlobes (Electro-cap International Inc., United States). The data was registered by the ERPrec software (BEE Medic GmbH, Switzerland) with a NeuroAmpx23^®^ amplifier and a sampling rate of 500 Hz. The impedance was kept below 5 kΩ. The signal was bandpass filtered between 0.5 and 50 Hz and down-sampled to 250 Hz. We changed the reference from linked-earlobe montage to average montage before preprocessing.

Raw EEG data were preprocessed and analyzed using in-house Matlab-based software. The data was band pass filtered between 0.5 and 50 Hz. We then applied an independent component analysis to remove eye blinks, horizontal eye movements and muscular artifacts recorded on Fp1, Fp2, T3, and T4 (Jung et al., 2000). To remove remaining artifacts (e.g., facial muscle activity), we used an automated pipeline rejecting segments with amplitudes above 100 μV and excessive activity in the 0–3 Hz and 20–30 Hz bands (threshold: channel *z*-score of 6). ERPs averaged over less than 40 valid (artifact-free, correct behavioral response) trials were not considered (the number of excluded subjects is listed in Supplementary Table 1).

**FIGURE 1:**
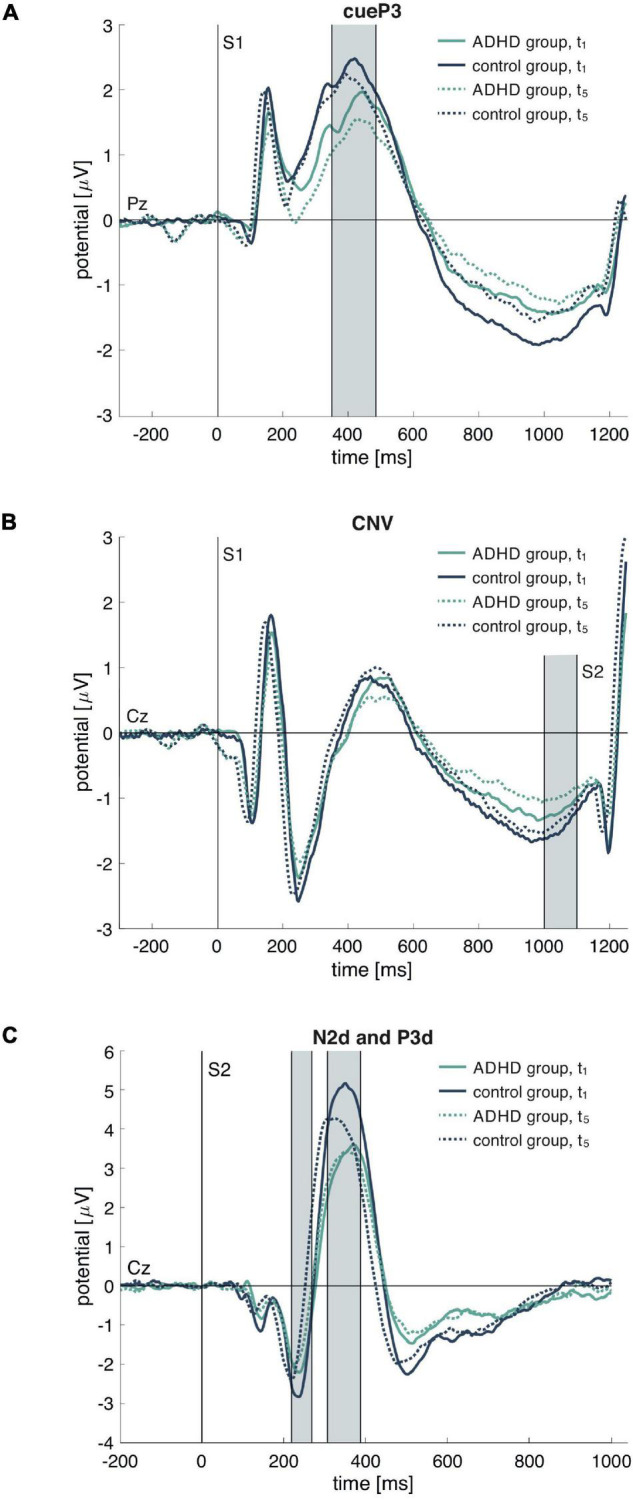
Grand average event-related potential (ERP) waves for the attention-deficit/hyperactivity disorder (ADHD) and control group in **(A)** all cue conditions (A–A or A–P) for cueP3 on Pz, **(B)** all cue conditions (A–A or A–P) for CNZ on Cz and **(C)** the difference curve between NoGo (A–P) and Go (A–A) after S2 for N2d and P3d. Displayed are the curves for the first (t_1_) and last (t_5_) assessment. The gray bars indicate analyzed time windows for the ERP extraction. S1 marks the cue stimulus and S2 the target stimulus.

The artifact-free data was further analyzed with a custom-built EEGlab plug-in. We applied baseline correction using a 100 ms pre-stimulus period (S1 for cueP3 and CNV, S2 for P3d and N2d). The peak amplitudes and latencies of the components cueP3, P3d, and N2d were determined within an adjusted time window based on the grand average curve (80% of the time interval between the peak of interest and the preceding peak). The windows of the analyzed ERP components are 348–484 ms after S1 onset for cueP3, 220–268 ms after S2 onset for N2d and 308–388 ms after S2 onset for P3d. For the individual peak detection within these windows, we applied self-modeling warping functions (Gervini and Gasser, 2004) to overcome the inter-individual temporal variability. The CNV amplitude was calculated by the mean voltage within the 100 ms time window before the onset of the second stimulus. Grand average curves of both groups for the first (t_1_) and last (t_5_) assessments are displayed in Figure 1.

### Statistical Analysis

First, we examined the group differences for the self-reported symptoms, behavioral, and ERP measures between the ADHD and control groups at baseline (cross-sectional). Second, we describe the average changes over time (longitudinal). These first two aims were addressed by latent growth curve models. As dependent variables, we used self-reported ADHD symptoms (inattention and hyperactivity), behavioral measures of the Go/NoGo task (reaction time, reaction time variability, omission, and commission errors), and ERP amplitudes and latencies at the assessment time points t_1_ to t_5_. We added group (control = 0, ADHD = 1), sex (male = 0, female = 1) and age at the first assessment (centered to the average age of the sample) as time invariant covariates on the intercept and slope estimates. For the longitudinal descriptions of the dependent variables, we described the change over time within the control group and the potential distinctive pattern observed in the ADHD group.

**FIGURE 2:**
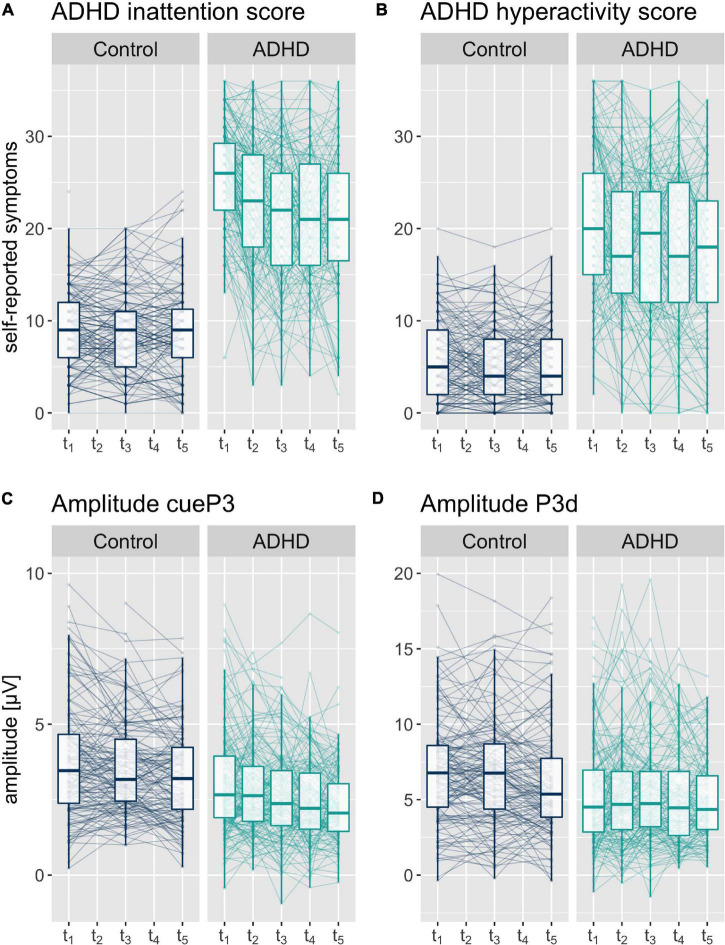
Boxplot with median and 25%-und 75% quantiles and individual values of self-reported symptoms **(A)** ADHD inattention and **(B)** ADHD hyperactivity sum score, and **(C)** cueP3 and **(D)** P3d amplitudes at the five assessment time points t_1_ to t_5_ (assessment interval approximately 6 months).

Third, we examined potential associations between the symptom burden and the objective measures of the Go/NoGo task. This third aim was addressed by bivariate structural equation models, including the scores of the self-reported symptoms and the behavioral or ERP measures. We were interested in the associations between the person-specific intercepts (cross-sectional) as well as between the person-specific slopes (change over time). We used the lavaan package version 0.6-5 (Rosseel, 2012) in R to specify the models. Models were estimated by the full information maximum likelihood approach, which provides unbiased estimates under the missing at random assumption. To account for the non-linear decrease in ADHD symptoms (see Figures 2A,B), we added an average quadratic term for ADHD inattention and ADHD hyperactivity. The associations between the self-reported symptoms and the other variables were based on the linear person-specific slopes. We used the guidelines by Schermelleh-Engel et al. (2003) to rate the goodness of fit and report the degrees of freedom, chi-square distribution (chisq), Root Mean Square Error of Approximation (rmsea) and the Comparative Fit Index (CFI) as fit measures. There was insufficient random slope variance in the bivariate models with the ADHD hyperactivity score for adequate model fitting. By setting the random slope variance of the ADHD hyperactivity score to zero we lost the possibility to compute correlations with the objective measures.

We report 95% confidence intervals for the model estimates. The reliability of a measurement is calculated by intraclass correlation coefficients (ICC) and categorized into low (<0.50), moderate (0.5–0.75), good (0.75–0.9), and excellent (>0.9) (Koo and Li, 2016). The reported ICCs are model based, hence adjusted for group, sex, and age. The ICCs are defined as the ratio of the between-participant variance to the total variance [random intercept/(random intercept + error)] (Bartlett and Frost, 2008).

## Results

### Symptoms and Attention-Deficit/Hyperactivity Disorder Presentation

Self-reported symptom burden was used to define the participants’ ADHD subtype. At the first assessment t_1_, *N* = 86 (41%) report inattentive, *N* = 11 (5%) hyperactive and *N* = 69 (33%) combined ADHD (inattentive and hyperactive). Data is missing for *N* = 10 (5%) and *N* = 34 (16%) did not fulfill the DSM-5 criteria based on their self-report. At the last assessment, t_5_ *N* = 28 (13%) report inattentive, *N* = 9 (5%) hyperactive and *N* = 29 (14%) comorbid ADHD symptoms. Data is missing for *N* = 80 (38%) and *N* = 64 (30%) did not fulfill the DSM-5 criteria based on their self-report. Details on the change in ADHD diagnosis and subtype are shown in Table 2. Overall, 56 participants are in the same clinical category at the beginning and the end of the trial. This corresponds to 43% when considering the subsample of participants with complete data for t_1_ and t_5_ (*N* = 126) and low agreement unweighted Cohen’s kappa (0.25 [0.15, 0.36]).

**TABLE 2:** Distribution of attention-deficit/hyperactivity disorder (ADHD) presentation at the first t_1_ and last t_5_ assessment based on self-reported symptom burden.

		First assessment t_1_					
		ADHD-com	ADHD-hyper	ADHD-inatt	non-ADHD	Missing	Total
**Last assessment t_5_**	**ADHD-com**	18	1	8	2	0	29
	**ADHD-hyper**	6	2	0	1	0	9
	**ADHD-inatt**	7	1	18	1	1	28
	**non-ADHD**	15	4	24	18	3	64
	**Missing**	23	3	36	12	6	80
	**Total**	69	11	86	34	10	210

*We categorized according to the DSM-5: combined presentation (ADHD-com), hyperactive presentation (ADHD-hyper) and inattentive presentation (ADHD-inatt). Participants, who did not fulfill the diagnostic criteria are categorized as non-ADHD. Participants who did not fill in the questionnaire (N = 10 t_1_, N = 80 t_5_) are summarized in the category missing.*

### Medication Intake and Therapies

The data about medication intake and attended therapies was collected only for the ADHD group (*N* = 210). All participants were medication-free at the day of the assessment. However, some took medications for their daily life. The intake of methylphenidate and antidepressants in the ADHD group decreased from the first to the last assessment [methylphenidate t_1_: *N* = 73 (35%), t_5_: *N* = 56 (27%) and antidepressants (t_1_: *N* = 39 (19%), t_5_: *N* = 32 (15%)]. Data of medication intake was missing for *N* = 54 (26%) at t_1_ and *N* = 77 (37%) at t_5_.

Attended therapies during the study comprised psychotherapy, neurofeedback, and other types of supportive trainings or consultations. The percentage of participants attending the different types of therapies remained constant over the two years [psychotherapy; t_1_ *N* = 36 (17%), t_5_: *N* = 41 (20%), neurofeedback; t_1_: *N* = 4 (2%), t_5_ *N* = 13 (6%) neurofeedback, others; t_1_: *N* = 35 (17%), t_5_: *N* = 34 (16%)]. Data of therapy attendance was missing for *N* = 47 (22%) at t_1_ and *N* = 76 (36%) at t_5_.

### Cross-Sectional Group Differences

The basic models in Table 3 address a male participant of the control group of average age (34 years). The effect of group (ADHD = 1) describes the deviation of the ADHD participants from the controls, on a cross-sectional level. The model fits of the univariate models are good or acceptable depending on the reported fit measures. Details on the fit measures are provided in the Supplementary Table 3.

**TABLE 3:** Univariate latent growth curve models describing group average values (intercepts) and average changes over time (slope) with 95% confidence interval.

	Intercept		Slope		ICC
	(value at the first assessment)		(change per assessment interval)		
	Control group	Effect of group	Control group	Effect of group	
**ADHD symptoms**					
ADHD inattention	9.3 [8.2, 10.5]	**16.3 [15.2, 17.5]**	−0.2 [−0.5, 0.2]	**−1.0 [−1.3, −0.6]**	0.61
ADHD hyperactivity	6.2 [4.8, 7.6]	**14.2 [12.8, 15.6]**	−0.30 [−0.62, 0.01]	**−0.7 [−1.0, −0.4]**	0.67
**Behavioral measures**					
RT	351 [333, 370]	**24 [5, 42]**	−3 [−7, 1]	−3.3 [−7, 1]	0.77
RTcv	20.24 [18.97, 21.52]	**3.65 [2.39, 4.91]**	−0.01 [−0.37, 0.35]	−0.01 [−0.36, 0.34]	0.58
Commission errors	0.50 [0.09, 0.91]	**0.52 [0.12, 0.92]**	−0.01 [−0.1, 0.08]	−0.04 [−0.14, 0.05]	0.69
Omission errors	1.38 [0.55, 2.2]	**1.81 [1, 2.62]**	0.27 [−0.07, 0.6]	0.12 [−0.21, 0.45]	0.34
**ERP amplitudes**					
cueP3	3.32 [2.97, 3.66]	**−0.49 [−0.84, −0.15]**	**−0.08 [−0.16, −0.01]**	−0.07 [−0.14, 0.01]	0.76
CNV	−1.45 [−1.65, −1.26]	**0.37 [0.18, 0.56]**	0.02 [−0.03, 0.08]	0.01 [−0.04, 0.07]	0.62
P3d	6.26 [5.49, 7.03]	**−1.31 [−2.07, −0.55]**	**−0.24 [−0.4, −0.08]**	**0.19 [0.03, 0.35]**	0.80
N2d	−3.17 [−3.67, −2.68]	**0.18 [−0.31, 0.67]**	**0.12 [0.01, 0.23]**	−0.02 [−0.12, 0.09]	0.70
**ERP latencies**					
cueP3	411.09 [397.94, 424.24]	**20 [7, 33]**	−3 [−6, 1]	3 [−1, 6]	0.57
P3d	353.20 [345.98, 360.41]	6 [−1, 13]	**−4 [−6, −2]**	2 [−1, 4]	0.53
N2d	245.07 [239.73, 250.4]	1 [−5, 6]	**−3 [−5, −5]**	2 [0, 3]	0.39

*The group effect describes the deviation of the ADHD group from the control group.*

*RT, reaction time in milliseconds; RTcv, coefficient of variance of reaction time (RTcv). The unit of the ERP variables are μV for the amplitudes and milliseconds for the latencies. Significant parameter estimate are shown in bold.*

At the first time point, the control group reported an overall ADHD inattention score of 9 and the ADHD group of 25. Further, the overall ADHD hyperactivity score was in general lower in the control group, with 6 compared to 20 in the ADHD group. Regarding the behavioral measures, the ADHD group showed an increased mean reaction time (24 ms [5, 42 ms]), reaction time variability (3.65 [2.39, 4.91]) and number of commission (0.52 [0.12, 0.92]) and omission (1.81 [1.00, 2.62]) errors. There is evidence that the cueP3, CNV, and P3d amplitude were smaller in the ADHD group compared to the control group, represented by negative coefficients for positive components (cueP3: −0.49 μV [−0.84, −0.15 μV], P3d: −1.31 μV [−2.07, −0.55 μV]) and positive coefficients for the negative components (CNV: 0.37 μV [0.18, 0.56 μV]). In addition, the latency of the cueP3 component was increased in the ADHD compared to the control group (20 ms [7, 33 ms]). There is absence of evidence for group differences in the N2d amplitude, the P3d and N2d latencies.

The complete models, including the effect of sex and age (variables of no interest) are described in the Supplementary Table 2. In addition, the effect sizes of the group differences are described in a previous publication (Münger et al., 2021) including a thorough discussion of the effect of potential moderating factors like IQ, medication intake and degree of symptom burden.

### Longitudinal Changes

The slope estimates of the basic models (Table 3) describe the average change from one assessment to the next for a male participant of the control group. The deviation in change over time of the ADHD group from the control group is defined by the slope estimate of group. Therefore, the average change from one assessment time point to the next for the ADHD group is obtained by adding the effect of group to the control group’s slope.

There was no evidence for a change over time in the self-reported ADHD symptoms in the control group (ADHD inattention: −0.2 [−0.5, 0.2], ADHD hyperactivity: −0.3 [−0.6, 0.0]). In contrast, for the ADHD group the self-reported symptoms decreased over time (ADHD inattention: −1.0 [−1.3, −0.6], ADHD hyperactivity: −0.7 [−1.0, −0.4]). Hence, the total decrease of self-reported symptoms per time-point in the ADHD group was −1.2 in the ADHD inattention score, and −1.0 in the ADHD hyperactivity score.

We did not observe any change over time in any of the behavioral measures in the control group, nor did we reveal evidence for a deviating pattern in the ADHD group for any of the behavioral measures.

In the ERP components, we obtained evidence for changes over time in the cueP3, P3d, and N2d amplitude, as well as the P3d and N2d latency. We observed a comparable change over time in the cueP3 amplitude in both groups. The amplitude of this component decreased by −0.08 μV [−0.16, −0.01 μV] in the control group. The model indicates a non-significant effect of group (−0.07 μV [−0.14, 0.01 μV]), indicating comparable decrease per time point, see also Figure 2C. The amplitude of the later P3d decreased in the control group by −0.24 μV [−0.40, −0.08 μV] per time point. The model indicates a positive effect of group of 0.19 μV [0.03, 0.35 μV], resulting in a smaller decrease in the ADHD group of only −0.05 μV, see also Figure 2D.

The amplitude of the N2d component attenuated by 0.12 μV [0.01, 0.23 μV] in the control group. The non-significant effect of group (−0.02 μV [−0.12, 0.09 μV]), indicates comparable decrease in both groups. We did not observe change over time in the CNV amplitude. There is no evidence for change in the cueP3 latency over time for the control group (−3 ms [−6, 1 ms]), nor evidence for an effect of group (3 [−1, 6]).

The latencies of the P3d and N2d component are decreasing in the control group over time (P3d: −4 ms [−6, −2 ms], N2d: 3 ms [−5, −2 ms]). However, there is no evidence for an effect of group for these two components (P3d: 2 ms [−1, 4 ms], N2d: 2 ms [0, 3 ms]).

To fathom the change over time of the P3d amplitude, we investigated the ERP components of the Go and NoGo conditions separately. The intercepts and group differences of the GoP3 and NoGoP3 amplitudes are comparable to those of the P3d component (GoP3: controls: 7.00 μV [6.34, 7.65 μV], group effect: −1.43 μV [−2.08, −0.79 μV], NoGoP3: 11.46 μV [10.42, 12.50 μV], group effect: −2.49 μV [−3.52, −1.46 μV]). The slope estimates and its effect of group reveal how these two components change over time within the control and ADHD group. The GoP3 amplitude tends to decrease over time in the control group (−0.17 μV [−0.32, −0.01 μV]). Although the negative group effect for ADHD (−0.06 μV [−0.21, 0.09 μV]) is non-significant, it is indicating a slightly stronger decrease in the ADHD group.

The NoGoP3 amplitude tends to decrease over time in the control group as well (−0.34 μV [−0.55, −0.13 μV]). Although the group effect of ADHD for this component is non-significant (0.11 μV [−0.10, 0.32 μV]), it is important to mention that this positive estimate points toward a weaker decrease in the ADHD group over time. To sum up, the total change per assessment in the control group is larger for the NoGoP3 amplitude (−0.34 μV) compared to the GoP3 amplitude (−0.17 μV). Therefore, the amplitude of the difference curve P3d (NoGoP3 minus GoP3) decreases over time in the control group (see Table 3). In contrast, the change per time-point in the ADHD group is similar for the NoGoP3 amplitude (−0.23 μV) compared to GoP3 (−0.23 μV). This comparable decrease explains the missing effect of time in P3d for the ADHD group (see Table 2). The results of the GoP3 and NoGoP3 latencies are comparable (GoP3: controls: −5 ms [−7, −2 ms], group effect: 5 ms [3, 8 ms], NoGoP3: −5 ms [−7, −3 ms], group effect: 5 ms [3, 7 ms]). These results indicate a decrease in the GoP3 and NoGoP3 latency in the control group. However, the group effect for ADHD is positive and of a similar magnitude as the change for the control group, indicating no change over time in the latencies for the ADHD group. For the sake of completeness, we also checked the association of the amplitudes and latencies of these two ERP components with the ADHD symptoms score. There is no evidence for any association.

### Associations Between Self-Reported Symptoms and Visual Continuous Performance Test Variables in Attention-Deficit/Hyperactivity Disorder: Cross-Sectional and Longitudinal

We fitted bivariate models to investigate the associations between the self-reported symptoms of the ADHD inattention and the ADHD hyperactivity score with the various behavioral and neurophysiological variables. The models were fitted within the ADHD group (*N* = 210 at t_1_). The correlation coefficients in Table 4 derive from the covariance of the person-specific intercepts and slopes in the bivariate models. The model fits of the bivariate models with the ADHD inattention score are acceptable and with the ADHD hyperactivity score good or acceptable (depending on the reported fit measure). Details of the fit measures of the bivariate models are provided in Supplementary Table 4.

**TABLE 4:** Correlation coefficients with 95% confidence intervals between self-reported symptoms (ADHDH inattention and ADHD hyperactivity) and the visual continuous performance test (VCPT) variables, behavioral measures and event-related potential (ERP) amplitudes and latencies.

	ADHD inattention		ADHD hyperactivity	
	Intercepts	Slopes	Intercepts	Slopes
**Behavioral measures**				
RT	−0.05 (−0.25, 0.15)	−0.17 (−0.55, 0.21)	0.07 (−0.10, 0.24)	–
RTcv	0.19 (−0.02, 0.40)	−0.38 (−0.97, 0.22)	**0.17 (0.00, 0.34)**	–
commission errors	**0.19 (0.00, 0.38)**	0.10 (−0.34, 0.53)	0.05 (−0.12, 0.21)	–
omission errors	0.22 (−0.02, 0.46)	0.15 (−0.33, 0.63)	0.16 (−0.02, 0.35)	–
**ERP amplitudes**				
cueP3	−0.13 (−0.32, 0.07)	−0.01 (−0.65, 0.64)	−0.03 (−0.20, 0.13)	–
CNV	0.00 (−0.21, 0.21)	0.10 (−0.35, 0.55)	−0.05 (−0.23, 0.12)	–
P3d	0.04 (−0.16, 0.24)	0.56 (−0.25, 1.38)	−0.09 (−0.26, 0.07)	–
N2d	0.01 (−0.20, 0.23)	0.84 (−0.35, 2.03)	−0.10 (−0.28, 0.07)	–
**ERP latencies**				
cueP3	**0.25 (0.01, 0.49)**	0.52 (−0.53, 1.57)	−0.05 (−0.24, 0.13)	–
P3d	0.06 (−0.14, 0.25)	–	−0.08 (−0.25, 0.09)	–
N2d	0.08 (−0.13, 0.29)	–	0.00 (−0.22, 0.22)	–

*The slope variance in some models was insufficient for a reliable parameter estimation and therefore set to zero (ADHD inattention: N2d and P3d latency; ADHD hyperactivity: all). Significant correlations are shown in bold.*

We cannot show any robust association between the self-reported symptom burden and the behavioral variables or ERP measures. On a cross-sectional level, we observed small positive correlations between ADHD inattention and the number of commission errors, as well as the cueP3 latency. For the ADHD hyperactivity score, we observed a small correlation with the reaction time variability. Of further note is that the confidence intervals are close to zero and many models were fitted.

There was no evidence for an association on a longitudinal level, hence there is no evidence for an association between the individual change in the ADHD symptoms scores and the behavioral or neurophysiological measures.

## Discussion

The current study characterizes a large sample of adults with ADHD in comparison to healthy controls. We replicated known group differences in the behavioral and ERP components from a classical cued Go/NoGo task on a cross-sectional level. Over the 2-year study period, we observed a reduction in self-reported symptoms within the ADHD group. The behavioral measures of neurocognitive performance, such as reaction time variability and number of errors, did not change over two years in any of the groups. For the ERP components, we observed a decrease of the cueP3 amplitude in both groups, and a decrease of the P3d amplitude in the control group.

Cross-sectionally, we found weak evidence for an association between the ADHD inattention symptoms and the number of commission errors, ADHD inattention symptoms and the P3d latency, and the ADHD hyperactivity symptoms and reaction time variability. Longitudinally, there is no evidence for any association between the changes in self-reported symptom burden and the investigated behavioral or neurophysiological measures from the cued Go/NoGo task.

### Attention-Deficit/Hyperactivity Disorder Symptom Burden and Presentation

The subjective symptom burden, expressed by the ADHD subjects in terms of perceived inattention and hyperactivity, decreased over the 2-year study period. These results are identical to those of Nylander et al. (2021), who also report a decrease in these subjective assessments, however, over five years. They reported symptom decrease in the self-report scales, as well as in the clinician assessments. In our cohort, the symptom decrease resulted in many participants who no longer fulfilled the DSM-5 criteria at the end of the study, which was not the case in the Nylander study (Nylander et al., 2021). In our sample, the symptom alleviation corroborates with the lower percentage of participants taking methylphenidate and antidepressants at the end of the study.

Furthermore, the symptom burden described in a categorical way, represented by the ADHD presentations, varied considerably from the begin to the end of the study. This adds to the low reliability of the categorizing diagnosis of ADHD discussed in children (Whitely, 2015). The symptom descriptions based on single DSM-5 criteria are neither particularly reliable. They are low and at best moderately. Symptoms of the hyperactivity domain are slightly more reliable than those of the inattention domain (Matte et al., 2015).

We did not expect such high alterations in the presentation and perceived burden of the ADHD symptoms among adults. Our results highlight the dynamic of the disorder – even during adulthood, and the urge to closely monitor ADHD patients of any age. The overall reduction in perceived symptoms is good news for the patients and proves that a combination of different therapeutic and lifestyle factors can alleviate the burden of the ADHD throughout the lifespan.

### Behavioral Measures

We replicated known group differences in the behavioral measures of the Go/NoGo task (Losier et al., 1996; Kofler et al., 2013; Grane et al., 2016; Hall et al., 2016; Münger et al., 2021). There is a large overlap between the clinical group and healthy controls, which results in small to moderate effect sizes. The behavioral measure with the largest effect size is reaction time variability, hence is most suitable to distinguish subjects with ADHD from controls (Kofler et al., 2013; Karalunas et al., 2014; Münger et al., 2021).

There was no change in the behavioral measures over the two years in our sample. The absence of performance improvement in the two groups allows two distinct inferences of the study’s results. Based on the lack of improvement in the control group we can exclude strong and conscious learning effects over the repeated assessments. The same result in the ADHD group further implies persisting cognitive deficits of the ADHD group on a behavioral level, despite the perceived symptom alleviation.

### Event-Related Potential Components

Similarly as in the behavioral measures, we replicated known cross-sectional group differences; namely attenuated cueP3, CNV, and P3d amplitudes in the ADHD group compared to the control group (Johnstone et al., 2013; Kaiser et al., 2020). The smaller amplitudes in individuals with ADHD supposedly represent lower attentional resources and cognitive capacities, potentially resulting in worse performance.

The cueP3 amplitude in both groups and the P3d amplitude in the control group decreased over two years. In this context, the attenuation can be interpreted as an habituation to the attention-attracting stimuli, such as the cue stimulus and an optimization of cognitive processes after the target stimuli. During habituation the involved neural network processes the incoming information and cognitive operations more efficiently. Several authors have argued that practice or habituation related increases in neural efficiency depends on neural or cognitive baseline activities (Neubauer et al., 2004; Kelly and Garavan, 2005). For example, the higher the IQ the larger the increase in neural efficiency during repeated processing. Our results partly support this idea since the control group demonstrates higher IQ scores than the ADHD group and had a stronger P3d amplitude decrease over two years.

Additional analyses revealed different decrease-patterns for the GoP3 and NoGoP3 between the two groups. In the ADHD group, these two components decreased similarly, whereas in the control group the NoGoP3 decreased more strongly compared to the GoP3, resulting in a more pronounced decrease of the P3d amplitude. Since the NoGoP3 amplitude represents the neural underpinnings of inhibition (Grane et al., 2016; Zarka et al., 2021) one might argue that the neuronal inhibition effort during NoGo trials diminished somewhat stronger for the control than for the ADHD group.

Comparison of baseline characteristics within the ADHD group revealed that subjects who were lost to follow-ups have larger baseline P3d amplitudes than subjects who completed the trial. Hence, the P3d amplitude of the individuals who dropped out was similar to the control group. This could have led to an overestimation of the group effect (ADHD participants in comparisons to controls).

The lack of change of the CNV amplitude over time indicates an absence of adaptation in the preparatory processes in both groups.

The results with respect of the ERP latencies are mixed. Cross-sectionally, we observed a longer cueP3 latency in the ADHD group compared to the control group. No group effect was seen in the P3d and N2d components. Longitudinally, we observed shorter N2d and P3d latencies at the final measurement in both groups reflecting faster stimulus processing over time.

In line with the results of the ERP amplitudes which indicate more efficient processing, the ERP latencies of the N2d and P3d components indicate swifter processing over time. The lack of longitudinal changes in the cueP3 latency indicates a lack of adaption in the speed of alertness related processing.

### Reliability of the Used Measures

To quantify the reliability of the self-reported symptom burden, behavioral, and ERP measures we computed model-based ICCs. In our sample the reliability of self-reported symptom burden scores, deriving from the DSM-5 items, is moderate to good. Likewise, the reliability of reaction time, reaction time variability, number of commission errors and ERP amplitudes is moderate to good, whereas the reliability of the ERP latencies is poor to moderate.

The short-term inter-individual reliability of ERP amplitudes were previously described to be moderate to high and the reliability of the latencies poor to moderate (Kompatsiari et al., 2016; Taylor et al., 2016; Behforuzi et al., 2019; Devos et al., 2020). Our analysis revealed generally lower reliability than reported in the literature. In comparison to other studies, we analyzed measurements spread over two years, during which actual neurophysiological alterations may have occurred. Furthermore, in contrast to previous studies, we computed model-based ICCs. As the ICC is highly dependent on the total variance in the sample, adjusting for potential group, age, and sex effects reduces the between-person variance in the model, which results in lower ICC scores.

Event-related potential latencies have generally lower ICCs in comparison with ERP amplitudes and behavioral CPT measures as observed previously by others (Kompatsiari et al., 2016; Taylor et al., 2016; Behforuzi et al., 2019; Devos et al., 2020). This is may partly due to methodological limitations of the routine latency computations (Kappenman and Luck, 2012; Luck and Gaspelin, 2017).

To sum up, we observed moderate reliability among ADHD symptom scores, reaction time, reaction time variability, number of commission errors and ERP amplitudes.

### Associations of Symptoms and Behavioral and Event-Related Potential Measures

On a cross-sectional level, there is weak evidence for an association of the number of reaction time variability, commission errors and cueP3 with ADHD symptom burden. However, these results should be interpreted with caution as the confidence interval boundaries are close to zero and the estimated correlation coefficients are small.

Other cross-sectional studies in adults with ADHD found small correlations between the symptom burden and individual ERP measures. Grane et al. (2016) used the same task as applied in the current manuscript and reported a significant negative correlation for GoP3, but not for cueP3, CNV, NoGoN2, or P3. Woltering et al. (2013) described a similar negative correlation between the inattention score and the P3 in another Go/NoGo task. Most likely, several studies that did not find evidence for correlations between the domains have not published the results of the correlation analysis. This kind of publication bias is well known in psychology and medicine studies (Kühberger et al., 2014; Van Aert et al., 2019).

On a longitudinal level, we did not find any evidence for correlations between the individual change in symptom burden and change in the behavioral or ERP measures. To our knowledge this is the first study among adults investigating the longitudinal association between symptom burden and executive functions, using both behavioral and neurophysiological measures. Recent developmental studies in ADHD similarly concluded that symptom alleviation was not associated with neurocognitive development (van Lieshout et al., 2019; Niina et al., 2021). Our results are in accordance with the liability hypothesis and suggest that the neurocognitive deficits and neurophysiological alterations are not mediated by symptom severity.

Furthermore, there are alternative explanations for the lack of evidence for an association between the symptom burden and neurophysiological measures. One possibility is the individually perceived cognitive load or experienced stress level during the task, especially in the cross-sectional analysis. Another explanation is the different levels of specificity that we look at: on one side the ERPs reflect very specific processes and on the other the side symptoms assess rather general difficulties in daily life. Altogether, our results are in line with previous research and corroborate the weak interdependency of the self-reported burden in daily life with the behavioral and neurophysiological measures. Apparently, the degree of symptom burden is not reflected in the magnitude of the objective measures for which research identified robust group differences between participants with ADHD and healthy controls. In addition, the absence of clear associations between the symptom burden and objective measures, both cross-sectionally and longitudinally, highlights the pivotal difficulties in psychiatric research. The field aims to establish more objective neurophysiological markers to describe deficient functioning, while being constrained by the clinical diagnosis that rely on the subjective perception of symptoms.

### Limitations

Several limitations apply to the study sample, the assessed clinical and demographical information, and performed statistical analysis.

First the dataset is heterogeneous in terms of age and ADHD presentation. Although limited from a strictly research-focused view, such large and heterogeneous samples are important from a clinical perspective. In addition, comorbidities were not assessed thoroughly. Similarly, we have only data available regarding methylphenidate and antidepressants intake. These medications, as well as others not assessed, may affected the results. Especially changes in the two years might have affected perceived symptom burden. Second, the symptom decrease resulted in a considerable number of participants who no longer fulfilled the DSM-5 criteria of ADHD at the end of the study. In addition, symptom burden and ADHD presentation are based on self-report of the individuals, hence are highly subjective and potentially biased. For example, non-credible responders tend to report higher rate of impairment than credible responders (Johnson and Suhr, 2021). Therefore, ascertainment of the diagnosis at each assessment would have reduced the uncertainty regarding the disorder severity. Furthermore, there is no information available, besides the behavioral measures during the cued Go/NoGo task, to understand the burden of ADHD. Third, in the ADHD group the subjects who completed the trial were on average older than those who were lost to follow-ups. This is in line with a retrospective analysis of a large sample about selectivity in longitudinal studies (Salthouse, 2014), in which the author identified higher return rates for older adults. Younger subjects may have more changes in their living conditions and are for example more likely lost due to moving away. Fourth, the use of ICC as a reliability measure is debated (Koo and Li, 2016), as it quantifies how well and individual can be discriminated from the population of interest. In addition, the ICC does not address the within-participant variability compared to the person-specific change over time, which would be important for precise estimation of longitudinal changes. Additional methodological limitations include an increased risk for type I error as we fitted many models for the same task.

## Conclusion

In conclusion, we have shown alteration of neuronal processing over two years, whereas neurocognitive performance at the behavioral level was constant. These adaptions are more pronounced in the control than in the ADHD group and potentially reflect improved neuronal efficiency. Using a relatively large sample of adults, we have shown that self-reported ADHD symptoms are not substantially related to objective measures of executive functioning neither cross-sectionally nor longitudinally. Furthermore, the decrease in symptom burden in this observational study and the low agreement on ADHD presentation between the first and last assessment reflects the low reliability of the ADHD diagnosis by clinical presentation.

## Data Availability Statement

The data tables and R analysis code will be made available on request. The raw EEG data are not publicly available and can only be accessed via collaborations with the Brain and Trauma Foundation Grisons.

## Ethics Statement

The studies involving human participants were reviewed and approved by Zurich’s cantonal ethics committee (LeitEKZH_2013 -0327/EKNZ_2014_160). The patients/participants provided their written informed consent to participate in this study.

## Author Contributions

GC, DE-H, and AM contributed to the design and data collection of the dataset. JK and HA-R contributed to the data collection. MM and SS performed the statistical analysis. MM wrote the first draft of the manuscript. MM, SS, GC, and LJ contributed to manuscript revision. All authors approved the submitted version.

## Conflict of Interest

The authors declare that the research was conducted in the absence of any commercial or financial relationships that could be construed as a potential conflict of interest.

## Publisher’s Note

All claims expressed in this article are solely those of the authors and do not necessarily represent those of their affiliated organizations, or those of the publisher, the editors and the reviewers. Any product that may be evaluated in this article, or claim that may be made by its manufacturer, is not guaranteed or endorsed by the publisher.
