# Supplementary material for: Longitudinal Analysis of Self-Reported Symptoms, Behavioral Measures, and Event-Related Potential Components of a Cued Go/NoGo Task in Adults With Attention-Deficit/Hyperactivity Disorder and Controls
Source: Front Hum Neurosci. 2022 Feb 18;16:767789. doi: 10.3389/fnhum.2022.767789 (PMC8894259; doi:10.3389/fnhum.2022.767789)
Supplement: Supplementary file 1 [file Table_1.docx]

Supplementary Table 1: Missing ERP data (participants with complete EEG assessment, but <40 valid trials per condition for the ERP computation).

|  | **t_1_** | | **t_2_** | | **t_3_** | | **t_4_** | | **t_5_** | |
| --- | --- | --- | --- | --- | --- | --- | --- | --- | --- | --- |
|  | control | ADHD | control | ADHD | control | ADHD | control | ADHD | control | ADHD |
| cue trials | 1 | 0 | NA | 1 | 3 | 2 | NA | 1 | 1 | 4 |
| Go trials | 0 | 3 | NA | 3 | 2 | 4 | NA | 1 | 0 | 6 |
| NoGo trials | 1 | 2 | NA | 4 | 4 | 5 | NA | 2 | 1 | 5 |
| difference curve | 1 | 3 | NA | 4 | 5 | 6 | NA | 3 | 1 | 10 |
