# Supplementary material for: Longitudinal Analysis of Self-Reported Symptoms, Behavioral Measures, and Event-Related Potential Components of a Cued Go/NoGo Task in Adults With Attention-Deficit/Hyperactivity Disorder and Controls
Source: Front Hum Neurosci. 2022 Feb 18;16:767789. doi: 10.3389/fnhum.2022.767789 (PMC8894259; doi:10.3389/fnhum.2022.767789)
Supplement: Supplementary file 2 [file Table_2.docx]

Supplementary Table 2: Complete univariate models, including the effect of sex and age (variables of no interest).

|  | **intercept**  (value at the first assessment) | | | | **slope**  (change per assessment interval) | | | |
| --- | --- | --- | --- | --- | --- | --- | --- | --- |
|  |  |  |  |  |  |  |  |  |
|  | control group | effect of group | effect of sex | effect of age | control group | effect of group | effect of sex | effect of age |
| **ADHD symptoms** | | | | | | | | |
| ADHD inattention | 9.3 [8.2, 10.5] | **16.3 [15.2, 17.5]** | -0.4 [-1.5, 0.7] | 0.0 [-0., 0.1] | -0.2 [-0.5, 0.2] | **-1.0 [-1.3, -0.6]** | 0.1 [-0.2, 0.5] | 0.0 [0.0, 0.0] |
| ADHD hyperactivity | 6.2 [4.8, 7.6] | **14.2 [12.8, 15.6]** | -0.5 [-1.9, 0.9] | **0.1 [0.0, 0.2]** | -0.3 [-0.6, 0.0] | **-0.7 [-1.0, -0.4]** | 0.3 [0.0, 0.6] | 0.0 [0.0, 0.0] |
| **Behavioral measures** | | | | | | | | |
| RT | 351 [333, 370] | **23.8 [5.4, 42.1]** | 15.3 [-2.9, 33.4] | **1.1 [0.3, 1.9]** | -2.7 [-6.8, 1.4] | -3.3 [-7.3, 0.7] | -3.3 [-7.3, 0.7] | 0.05 [-0.1, 0.2] |
| RTcv | 20.24 [18.97, 21.52] | **3.65 [2.39, 4.91]** | 0.48 [-0.76, 1.72] | **-0.07 [-0.12, -0.01]** | -0.01 [-0.37, 0.35] | -0.01 [-0.36, 0.34] | -0.16 [-0.51, 0.19] | 0.00 [-0.02, 0.01] |
| commission errors | 0.50 [0.09, 0.91] | **0.52 [0.12, 0.92]** | -0.03 [-0.43, 0.37] | 0.00 [-0.02, 0.01] | -0.01 [-0.1, 0.08] | -0.04 [-0.14, 0.05] | -0.01 [-0.1, 0.08] | 0.00 [-0.01, 0] |
| omission errors | 1.38 [0.55, 2.2] | **1.81 [1, 2.62]** | 0.20 [-0.6, 1] | 0.00 [-0.04, 0.03] | 0.27 [-0.07, 0.6] | 0.12 [-0.21, 0.45] | -0.23 [-0.55, 0.1] | 0.00 [-0.02, 0.01] |
| **ERP amplitudes** | | | | | | | | |
| cueP3 | 3.32 [2.97, 3.66] | **-0.49 [-0.84, -0.15]** | **0.54 [0.2, 0.88]** | **-0.06 [-0.07, -0.04]** | **-0.08 [-0.16, -0.01]** | -0.07 [-0.14, 0.01] | -0.04 [-0.12, 0.03] | 0.01 [0, 0.01] |
| CNV | -1.47 [-1.66, -1.27] | **0.37 [0.18, 0.56]** | -0.04 [-0.23, 0.15] | -0.01 [-0.02, 0] | 0.03 [-0.02, 0.09] | 0.01 [-0.04, 0.07] | 0.01 [-0.04, 0.07] | 0.00 [0, 0] |
| P3d | 6.26 [5.49, 7.03] | **-1.31 [-2.07, -0.55]** | **1.17 [0.41, 1.92]** | **-0.04 [-0.08, -0.01]** | **-0.24 [-0.4, -0.08]** | **0.19 [0.03, 0.35]** | 0.03 [-0.13, 0.19] | -0.01 [-0.01, 0] |
| N2d | -3.17 [-3.67, -2.68] | **0.18 [-0.31, 0.67]** | **-1.13 [-1.62, -0.64]** | **0.09 [0.07, 0.12]** | 0.12 [0.01, 0.23] | -0.02 [-0.12, 0.09] | 0.00 [-0.1, 0.11] | -0.01 [-0.01, 0] |
| **ERP latencies** | | | | | | | | |
| cueP3 | 411 [398, 424.24] | **20.3 [7.3, 33.2]** | -6.2 [-19.1, 6.6] | **1.5 [0.9, 2.1]** | -2.5 [-6.0, 1.1] | 2.7 [-0.8, 6.2] | 0.1 [-3.3, 3.5] | -0.1 [-0.3, 0.0] |
| P3d | 353 [346, 360] | 6.3 [-0.8, 13.4] | 0.4 [-6.6, 7.4] | **0.9 [0.6, 1.2]** | **-4.0 [-6.2, -1.8]** | 1.6 [-0.5, 3.8] | 1.0 [-1.1, 3.2] | **0.1 [0.0, 0.2]** |
| N2d | 245 [240, 250] | 0.6 [-4.7, 5.8] | -3.3 [-8.5, 1.8] | **0.4 [0.2, 0.6]** | **-3.2 [-4.9, -1.5]** | 1.7 [0.0, 3.4] | 0.1 [-1.5, 1.8] | **0.1 [0.0, 0.2]** |

*Note: Intercepts and slopes with 95% confidence interval. The categorical variables are coded as group; control=0, ADHD=1 and sex; male=0, female=1. Age represents the effect of every year deviating from the sample’s mean age (34 years). Abbreviations: RT; reaction time in milliseconds, RTcv; coefficient of variance of reaction time (RTcv=RTsd/RTmean). The unit of the ERP variables are µV for the amplitudes and milliseconds for the latencies*
