# Supplementary material for: Longitudinal Analysis of Self-Reported Symptoms, Behavioral Measures, and Event-Related Potential Components of a Cued Go/NoGo Task in Adults With Attention-Deficit/Hyperactivity Disorder and Controls
Source: Front Hum Neurosci. 2022 Feb 18;16:767789. doi: 10.3389/fnhum.2022.767789 (PMC8894259; doi:10.3389/fnhum.2022.767789)
Supplement: Supplementary file 3 [file Table_3.docx]

Supplementary Table 3: Measures of fit for the univariate models.

|  | df | chisq | rmsea | cfi |
| --- | --- | --- | --- | --- |
| **Self-reported symptoms** | | | | |
| ADHD inattention | 15 | 28 | 0.049 | 0.990 |
| ADHD hyperactivity | 15 | 12 | 0 | 1 |
| **Behavioral Measures** | | | | |
| RT | 19 | 35 | 0.048 | 0.982 |
| RTcv | 19 | 30 | 0.040 | 0.979 |
| commission errors | 19 | 45 | 0.061 | 0.955 |
| omission errors | 19 | 39 | 0.054 | 0.950 |
| **ERP amplitudes** | | | | |
| cueP3 | 19 | 22 | 0.022 | 0.997 |
| CNV | 19 | 36 | 0.050 | 0.966 |
| P3d | 19 | 33 | 0.045 | 0.985 |
| N2d | 19 | 25 | 0.028 | 0.993 |
| **ERP latencies** | | | | |
| cueP3 | 19 | 34 | 0.047 | 0.970 |
| P3d | 19 | 24 | 0.028 | 0.990 |
| N2d | 19 | 43 | 0.059 | 0.921 |

*Abbreviations: degrees of freedom (df), chi-square distribution (chisq), Root Mean Square Error of Approximation (rmsea), Comparative Fit Index (cfi) as fit measures.*
