# Supplementary material for: Longitudinal Analysis of Self-Reported Symptoms, Behavioral Measures, and Event-Related Potential Components of a Cued Go/NoGo Task in Adults With Attention-Deficit/Hyperactivity Disorder and Controls
Source: Front Hum Neurosci. 2022 Feb 18;16:767789. doi: 10.3389/fnhum.2022.767789 (PMC8894259; doi:10.3389/fnhum.2022.767789)
Supplement: Supplementary file 4 [file Table_4.docx]

Supplementary Table 4: Measures of fit for the bivariate models.

|  | **ADHD inattention** | | | | **ADHD hyperactivity** | | | |
| --- | --- | --- | --- | --- | --- | --- | --- | --- |
|  | df | chisq | rmsea | cfi | df | chisq | rmsea | cfi |
| **Behavioral Measures** | | | | | | | | |
| RT | 46 | 100 | 0.075 | 0.943 | 49 | 82 | 0.056 | 0.970 |
| RTcv | 45 | 85 | 0.065 | 0.945 | 49 | 65 | 0.039 | 0.982 |
| commission errors | 45 | 87 | 0.067 | 0.946 | 49 | 73 | 0.049 | 0.973 |
| omission errors | 45 | 94 | 0.072 | 0.919 | 49 | 78 | 0.053 | 0.962 |
| **ERP amplitudes** | | | | | | | | |
| cueP3 | 45 | 72 | 0.054 | 0.971 | 49 | 57 | 0.028 | 0.993 |
| CNV | 45 | 85 | 0.065 | 0.942 | 49 | 60 | 0.032 | 0.987 |
| P3d | 45 | 75 | 0.057 | 0.969 | 49 | 66 | 0.04 | 0.985 |
| N2d | 45 | 96 | 0.073 | 0.941 | 49 | 68 | 0.042 | 0.981 |
| **ERP latencies** | | | | | | | | |
| cueP3 | 45 | 92 | 0.071 | 0.927 | 49 | 74 | 0.050 | 0.968 |
| P3d | 48 | 77 | 0.054 | 0.959 | 49 | 64 | 0.038 | 0.983 |
| N2d | 48 | 86 | 0.061 | 0.930 | 49 | 79 | 0.054 | 0.956 |

*Abbreviations: degrees of freedom (df), chi-square distribution (chisq), Root Mean Square Error of Approximation (rmsea), Comparative Fit Index (cfi) as fit measures.*
