# Supplementary material for: Longitudinal Analysis of Self-Reported Symptoms, Behavioral Measures, and Event-Related Potential Components of a Cued Go/NoGo Task in Adults With Attention-Deficit/Hyperactivity Disorder and Controls
Source: Front Hum Neurosci. 2022 Feb 18;16:767789. doi: 10.3389/fnhum.2022.767789 (PMC8894259; doi:10.3389/fnhum.2022.767789)
Supplement: Supplementary file 5 [file Table_5.docx]

Supplementary Table 5a: Comparison of the baseline values (t_1_) between subjects who completed the trial and those who were lost to follow-up of all *categorical variables*. Reported are the number of individuals, its percentage from the total sample, and the p-value of the group comparison (completed vs. dropout) by a Fisher’s exact test. There is no data available for the control group for medication intake and attended therapied.

|  | **ADHD group, N=210** | | | **Control group, N=158** | | |
| --- | --- | --- | --- | --- | --- | --- |
|  | **completed**, N=148 | **dropout**, N=62 | **p-value** | **completed**, N=133 | **dropout**, N=25 | **p-value** |
| **sex** | | | 0.45 |  | | 0.20 |
| m | 78 (53%) | 29 (47%) |  | 39 (29%) | 11 (44%) |  |
| f | 70 (47%) | 33 (53%) |  | 94 (71%) | 14 (56%) |  |
| **methylphenidate intake** | | | 0.85 |  |  |  |
| no | 63 (44%) | 20 (43%) |  |  |  |  |
| yes | 56 (39%) | 17 (37%) |  |  |  |  |
| missing | 23 (16%) | 9 (20%) |  |  |  |  |
| **antidepressant intake** | | | 0.32 |  |  |  |
| no | 86 (61%) | 31 (67%) |  |  |  |  |
| yes | 33 (23%) | 6 (13%) |  |  |  |  |
| missing | 23 (16%) | 9 (20%) |  |  |  |  |
| **psychotherapy** | | | 0.90 |  |  |  |
| no | 96 (68%) | 31 (67%) |  |  |  |  |
| yes | 28 (20%) | 8 (17%) |  |  |  |  |
| missing | 18 (13%) | 7 (15%) |  |  |  |  |
| **neurofeedback** | | | 0.84 |  |  |  |
| no | 121 (85%) | 38 (83%) |  |  |  |  |
| yes | 3 (2.1%) | 1 (2.2%) |  |  |  |  |
| missing | 18 (13%) | 7 (15%) |  |  |  |  |
| **other therapies** | | | 0.68 |  |  |  |
| no | 99 (70%) | 29 (63%) |  |  |  |  |
| yes | 25 (18%) | 10 (22%) |  |  |  |  |
| missing | 18 (13%) | 7 (15%) |  |  |  |  |

Supplementary Table 5b: Comparison of the baseline values (t_1_) between subjects who completed the trial, and those who were lost to follow-up of all *continuous variables.* Reported are the mean, standard deviation, and the p-value of the group comparison (completed vs. dropout) by a t-test, significant results are marked in bold.

|  | **ADHD group, N = 210** | | | **control group, N = 158** | | |
| --- | --- | --- | --- | --- | --- | --- |
|  | **completed**, N=148 | **dropout**, N=62 | **p-value** | **completed**, N=133 | **dropout**, N=25 | **p-value** |
| **Demographic information** | | | |  |  |  |
| age | 36 (10) | 32 (9) | **<0.01** | 33 (12) | 29 (11) | 0.10 |
| IQ | 101 (15) | 96 (16) | 0.07 | 106 (14) | 105 (13) | 0.81 |
| **ADHD symptoms** | | | | | | |
| ADHD inattention | 25.8 (5.5) | 25.5 (5.4) | 0.74 | 9.2 (4.0) | 8.7 (5.4) | 0.70 |
| ADHD hyperactivity | 21 (8) | 19 (8) | 0.10 | 5.9 (4.4) | 5.5 (4.4) | 0.66 |
| **Behavioral Measures** | | | |  |  |  |
| RT | 396 (96) | 369 (104) | 0.09 | 359 (79) | 368 (89) | 0.65 |
| RTcv | 24 (7) | 24 (7) | 0.64 | 20.9 (5.3) | 21.0 (5.4) | 0.87 |
| commission errors | 3.4 (4.9) | 3.2 (4.6) | 0.79 | 0.50 (1.06) | 0.68 (1.18) | 0.25 |
| omission errors | 1.09 (2.77) | 1.21 (2.14) | 0.75 | 1.53 (2.61) | 1.04 (1.79) | 0.25 |
| **ERP amplitudes** | | | | | | |
| cueP3 | 2.90 (1.68) | 3.32 (1.63) | 0.09 | 3.82 (2.11) | 3.49 (1.72) | 0.40 |
| CNV | -1.17 (0.91) | -1.24 (0.96) | 0.62 | -1.49 (0.97) | -1.37 (1.00) | 0.59 |
| N2d | -3.44 (2.55) | -3.65 (2.28) | 0.55 | -3.96 (2.98) | -4.21 (2.70) | 0.68 |
| P3d | 5.0 (3.5) | 6.2 (3.4) | **0.02** | 7.1 (3.8) | 6.9 (3.6) | 0.78 |
| **ERP latencies** | | | | | | |
| cueP3 | 434 (64) | 414 (74) | 0.07 | 404 (69) | 394 (62) | 0.47 |
| N2d | 245 (29) | 240 (27) | 0.26 | 240 (25) | 232 (26) | 0.17 |
| P3d | 364 (38) | 349 (37) | **<0.01** | 350 (35) | 344 (36) | 0.45 |
